# Avacopan for severe pulmonary hemorrhage requiring extracorporeal membrane oxygenation in a patient with MPO-ANCA positive vasculitis

**DOI:** 10.1007/s13730-025-01068-0

**Published:** 2026-02-03

**Authors:** Keita Endo, Koichi Hayashi, Yuki Hara, Akihiro Miyake, Keisuke Takano, Kaede Yoshino, Koichi Kitamura, Shinsuke Ito, Shigeki Fujitani, Toshihiko Suzuki

**Affiliations:** 1 Department of Nephrology, Endocrinology & Diabetes, Tokyo Bay Urayasu Ichikawa Medical Center, 3-4-32, Todaijima, Urayasu, Chiba, 279-0001 Japan; 2 Department of Emergency and Critical Care Medicine, St Marianna University School of Medicine, Kawasaki, Japan

**Keywords:** Microscopic polyangiitis, ANCA-associated vasculitis, Avacopan, Prednisolone, Pulmonary hemorrhage, Extracorporeal membrane oxygenation

## Abstract

Myeloperoxidase-antineutrophil cytoplasmic antibody (MPO-ANCA)-positive vasculitis frequently affects the kidney and the lung, with alveolar hemorrhage being fatal. Whereas aggressive immunosuppressive therapies are conventionally used, recent studies have shown the beneficial effect of avacopan, a C5a antagonist, as an alternative to glucocorticoids for ANCA-associated vasculitis (AAV). In patients with pulmonary hemorrhage and severe respiratory failure, however, neither the efficacy of avacopan nor the contribution of this drug to early withdrawal of glucocorticoids is fully qualified. Here, we report a case of AAV presenting with alveolar hemorrhage requiring aggressive ventilatory support in which we experienced the favorable effect of early use of avacopan. A 31-year-old man was referred to our hospital because of a two-week history of blood sputum and positive MPO-ANCA. His respiratory failure deteriorated rapidly, necessitating both mechanical ventilation and extracorporeal membrane oxygenation. A combination therapy with glucocorticoids and rituximab was initiated and avacopan was started on hospital day 8, which resulted in successful remission within six months of admission (Birmingham Vasculitis Activity Score version 3 = 0), and the beneficial effect was sustained for at least 6 months following the discontinuation of glucocorticoid withdrawal (day 156). Thus, avacopan, in combination with immunosuppressives, may not only help suppress the disease activity of AAV but also facilitate early withdrawal of glucocorticoids even in case of life-threatening respiratory failure.

## Introduction

Antineutrophil cytoplasmic antibody (ANCA)-associated vasculitis (AAV) is a group of diseases characterized by inflammation of small to medium-sized blood vessels. Among the subtypes of AAV, myeloperoxidase (MPO)-ANCA-associated vasculitis frequently involves the lung and the kidney and causes serious outcomes, including acute kidney injury and diffuse alveolar hemorrhage, both of which are life-threatening complications. Traditionally, AAV with alveolar hemorrhage is recognized as the most severe form of this disease and the management of this requires aggressive immunosuppressive therapies, including high-dose glucocorticoids, cyclophosphamide and rituximab. These treatments, however, may cause severe adverse effects such as serious infections and bone marrow suppression, which hence arouses absolute needs for developing effective targeted therapies with less untoward effects.

Avacopan, a selective inhibitor of the C5a receptor, represents a novel therapeutic approach in the treatment of AAV. By blocking the C5a receptor, avacopan interferes with the complement pathway, a crucial role in the pathogenesis of AAV. Recently, the ADVOCATE trial has demonstrated the efficacy and safety of avacopan in inducing and maintaining remission in patients with AAV and has suggested its potential as an alternative to a glucocorticoid therapy [1]. This trial, however, did not evaluate severe cases with alveolar hemorrhage requiring mechanical ventilation and extracorporeal membrane oxygenation (ECMO). Furthermore, there have been published very few reports showing the use of avacopan as an induction therapy in such cases [2, 3]. Of note, only one study reported the use of avacopan alongside the implementation of ECMO, in which the patient was still on a considerable dose of prednisolone (25 mg/day) one year after the onset [2]. Thus, it remains unclear whether early withdrawal of prednisolone is feasible in cases of AAV complicated by severe respiratory failure requiring ECMO support.

Here, we present a patient with MPO-ANCA-associated vasculitis who manifested severe diffuse alveolar hemorrhage requiring ECMO support but acquired a sustained remission with avacopan alongside standard immunosuppressive treatment, with successful discontinuation of prednisolone within six months. This case highlights a potential of avacopan as an early therapeutic option in the management of life-threatening pulmonary complications associated with AAV.

## Case report

A 31-year-old man with no history of kidney disease was referred to our hospital for examination and treatment of MPO-ANCA associated vasculitis and respiratory failure. He had an annual medical checkup for the last 3 years and had a history of atopic dermatitis without use of medication. He had no other past history, family history or allergies.

Two months before admission, he experienced systemic joint pain and general fatigue and was treated as having the common cold by his family physician. At 1 week before admission, he had hemoptysis lasting 1 week and visited a hospital. Blood and urine tests revealed a normal level of serum creatinine (0.78 mg/dl) but elevated CRP (7.0 mg/dL) and abnormal urine (hematuria 2+, proteinuria 2+). Furthermore, positive MPO-ANCA and pulmonary infiltrate on computed tomography (CT) scan were observed, and hence he was referred to our hospital for emergency admission.

**Fig. 1:**
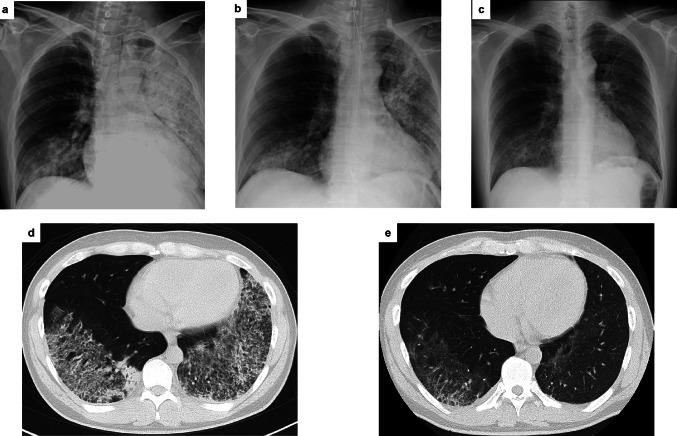
Temporal changes in pulmonary infiltration. Serial chest X-rays on the day of admission (**a**) and day 7 (**b**) in the supine position, and on day 12 (**c**) in the upright position. Serial CT images obtained on the day of admission (**d**) and day 14 (**e**)

Upon admission, the patient’s vital signs showed slightly elevated blood pressure (149/91 mmHg) and body temperature (37.9 ℃) and marked hyperpnea (respiratory rate = 29 breaths/min) was observed under 3L oxygen supplementation through canula; SpO_2_ was 99%. Physical examination revealed no uveitis, tenderness on cheek nor heart murmur. On auscultation, Fine crackles were heard over bilateral lung bases. There was no edema or skin rash in the extremities. Electrocardiogram showed no significant abnormal findings. CT scan of the lung showed diffuse bilateral infiltrate with ground-glass opacities (Fig. 1). Urinalysis indicated hematuria and proteinuria, and other laboratory data revealed marked anemia (Hb 7.7 g/dL) and elevated CRP (22 mg/dL) and MPO-ANCA (67 U/mL) (Table 1). Based on these data, we diagnosed the patient as having MPO-ANCA associated vasculitis affecting systemic organs, including the kidney and the lung. Birmingham Vasculitis Activity Score version 3 (BVAS-3) was 18 (6 points for chest (respiratory failure), 12 points for renal (hypertension, proteinuria > 1 g/day, hematuria > 10/HPF)).

**Table 1:** Laboratory data on admission

*Complete blood cell count*		
WBC	8800	/μL
RBC	260	×10^4^/μL
Hemoglobin	7.7	g/dL
MCV	88.1	fL
Platelet	29.3	×10^4^/μL
*Blood coagulation*		
aPTT	33.9	sec
PT-INR	1.32	
Fibrinogen	639	mg/dL
*Chemistry*		
Total protein	5.7	g/dL
Albumin	2.7	g/dL
UN	7.8	mg/dL
Creatinine	0.78	mg/dL
Uric Acid	2.7	mg/dL
AST	14	U/L
ALT	19	U/L
ALP	65	U/L
γ-GT	25	U/L
LD	237	U/L
CK	45	U/L
HDL-C	34	mg/dL
LDL-C	94	mg/dL
TG	63	mg/dL
Glucose	99	mg/dL
Hemoglobin A1c	5.8	%
Na	141	mEq/+
K	3.5	mEq/L
Cl	101	mEq/L
Ca	8.4	mg/dL
P	3.2	mg/dL
CRP	22.0	mg/dL
IgG	1066	mg/dL
IgA	114	mg/dL
IgM	29	mg/dL
C3	135	mg/dL
C4	28	mg/dL
CH_50_	45	U/mL
β2-MG	1.67	mg/L
Anti-nuclear antibody	160	
Anti-DNA antibody	Negative	
Anti-Sm antibody	<0.7	
Anti-SS-A antibody	86.5	U/mL
Anti-SS-B antibody	Negative	
MPO-ANCA	67	U/mL
PR3-ANCA	<1.0	U/mL
Anti-GBM antibody	<1.5	U/mL
Cryoglobulin	Negative	
KL-6	264	U/mL
*Urine analysis*		
pH	6.5	
Specific gravity	1.008	
RBC	30-49	/HPF
WBC	1-4	/HPF
Protein/creatinine	0.91	g/gCr

*WBC* white blood cells; *RBC* red blood cells; *MCV* mean corpuscular volume; *aPTT* activated partial thromboplastin time; *UN* urea nitrogen; *AST* aspartate aminotransferase; *ALT* alanine aminotransferase; *ALP* alkaline phosphatase; *LD* lactate dehydrogenase; *CK* creatine kinase; *HDL* high density lipoprotein cholesterol; *LDL* low density lipoprotein cholesterol; *TG* triglyceride; *CRP* C-reactive protein; *IgG *immunoglobulin G; *IgA* immunoglobulin A; *IgM* immunoglobulin M; *C3* complement component 3; *C4* complement component 4; *MPO-ANCA*, myeloperoxidase-anti-neutrophil cytoplasmic antibody; *β2-MG* beta2 microglobulin; *GBM* glomerular basement membrane; HPF high power field

### Clinical course

**Fig. 2:**
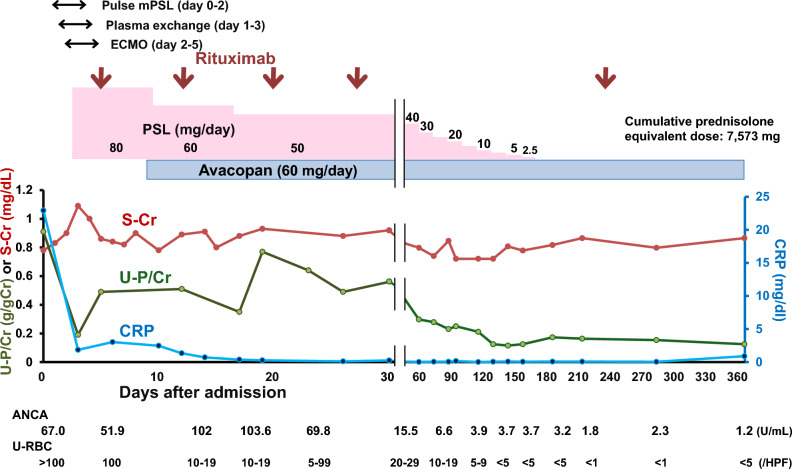
Clinical course and prednisolone dosage. Pulse methylprednisolone was administered from the day of admission to day 2. Plasma exchange was performed from day 1 to day 3. The patient required ECMO from day 2 to day 5. Rituximab was administered on days 5, 12, 20, 27, and 212. Avacopan was initiated on day 8. A kidney biopsy was performed on day 16. The patient was discharged on day 31. After discharge, he remained in remission even after steroid discontinuation. mPSL methylprednisolone; PSL prednisolone; ECMO extracorporeal membrane oxygenation; CRP C- reactive protein; S-Cr serum creatinine; Urine Proteinss/creatinine U-P/Cr; ANCA Antineutrophil cytoplasmic antibody; Urine red blood cells U-RBC

On the day of admission, a steroid pulse therapy (methylprednisolone; 1 g/day for 3 days) was started due to intractable hypoxemia secondary to diffuse alveolar hemorrhage (Figs. 1, 2). On hospital day 1, plasma exchange was initiated and was continued for a total of three days until the patient was confirmed negative for anti-glomerular basement membrane antibodies. On hospital day 2, the patient was intubated due to worsening respiratory failure. Hypoxemia was progressed (SpO_2_ = 90%), however, despite maximal FiO_2_ (i.e., 1.0), and hence veno-venous ECMO was implemented. At that time, a bronchoalveolar lavage confirmed the diagnosis of alveolar hemorrhage.

**Fig. 3:**
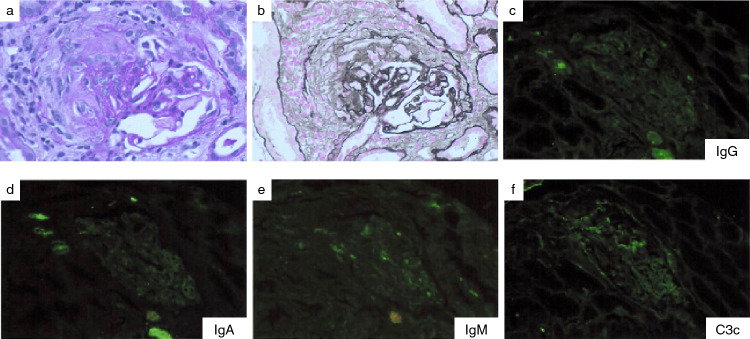
Kidney biopsy specimen. Light microscopy shows a cellular crescent in the glomeruli (**a** periodic acid-Schiff stain; **b** periodic acid-methenamine-silver stain). No significant immunofluorescence staining for IgG (**c**), IgA (**d**), IgM (**e**), or C3c (**f**) was detected

On hospital day 5, rituximab was added on the prednisolone therapy, at which time his respiratory status slightly improved (Fig. 1). Hence, he was weaned off ECMO support and extubated on hospital day 7 while avacopan (30 mg twice daily) was started on hospital day 8. A renal biopsy was performed on hospital day 16. Among 14 glomeruli, one showed global sclerosis and another segmental sclerosis. Of the remaining 12 glomeruli, one manifested a fibrous crescent (Fig. 3) and another exhibited glomerular segmental necrosis. No glomeruli showed mesangial proliferation. Tubular atrophy and tubulointerstitial fibrosis with inflammatory cell infiltrates were observed. No evidence of arteritis was observed in the interlobular arteries. Immunofluorescent analysis showed no significant deposition of immunoglobulins or complement components. Electron microscopy revealed no electron deposits. These histological findings were suggestive of pauci-immune crescentic glomerulonephritis.

The lung infiltrate observed on CT scan was improved (Fig. 1) and hemoptysis ceased by hospital day 24. He received four weekly rituximab injections and was discharged on hospital day 31, whereupon the dose of prednisolone was reduced to 40 mg/day (Fig. 2). At the first visit after discharge, there were no signs of relapse, and both the MPO-ANCA titer and the BVAS-3 were improved (MPO-ANCA, from 69.8 to 15.5 U/mL; BVAS-3, from 18 to 5 points). BVAS-3 decreased to zero on day 142. Prednisolone was gradually tapered and was completely withdrawn on day 156 (cumulative prednisolone equivalent dose: 7573 mg, including pulse methylprednisolone), but no exacerbation of the disease activity was observed thereafter. The glucocorticoid toxicity index-cumulative worsening score (GTI-CWS) showed zero value on day 93 and GTI-aggregate improvement score (GTI-AIS) from baseline was increased to + 10 on day 184.

## Discussion

ANCA-associated vasculitis damages small and medium-sized blood vessels of multiple vital organs, and its subtype, microscopic polyangiitis (MPA), frequently involves renal and pulmonary lesions. The present case, manifesting both renal (rapidly progressive glomerulonephritis) and lung involvement (alveolar hemorrhage or interstitial pneumonia), was compatible with definite MPA, based on the criteria established by Japanese Ministry of Health, Labour and Welfare. More importantly, the comprehensive therapy, including immunosuppressive drugs (steroids, rituximab), ECMO support and early treatment with avacopan, could rescue the patient from this potentially fatal disease with severe respiratory failure and lead to remission with no maintenance steroid therapy.

There has been great interest in the role of the complement pathway in exacerbating vascular inflammation in AAV. Thus, inflammatory cytokines and C5a produced by infection or other causes act on neutrophils, causing them to express their corresponding antigens (MPO or proteinase-3) on the plasma membrane [4]. Then, ANCA binds to them, resulting in overactivation of neutrophils and vascular endothelial damage. Avacopan, a selective inhibitor of the C5a receptor, suppresses this process and is demonstrated to offer favorable impacts on the progression of AAV [1–3, 5–8]. In the ADVOCATE trial, however, the patients with alveolar hemorrhage requiring invasive pulmonary ventilation were not enrolled [1] while our case with diffuse alveolar hemorrhage manifested severe respiratory failure requiring mechanical ventilation and ECMO support. Retrospective studies in patients with alveolar hemorrhage necessitating ventilation support also showed that early treatment with avacopan in addition to standard immunosuppressive treatment resulted in amelioration of alveolar hemorrhage and suggested its effectiveness as an additional promising strategy for severe alveolar hemorrhage [2, 3]. Although the respiratory status of our patient tended to improve when avacopan was started, there was no subsequent relapse of the disease and the lung infiltrates rapidly disappeared without any adverse events. In Japan, alveolar hemorrhage was present in 15.4% of the patients with AAV, of which 94% were MPO-ANCA positive [9]. Since alveolar hemorrhage is the leading cause of death within the first month of the onset [10], timely therapeutic intervention is crucial. It warrants further studies whether the inclusion of avacopan in the induction treatment of AAV may facilitate the mitigation of pulmonary involvement [3].

Whilst prolonged glucocorticoid treatment involves adverse effects, recent studies suggest that avacopan may allow rapid tapering of steroid administration and possess a steroid-sparing effect [2, 11]. Furthermore, the timing of avacopan administration is reported to be associated with the cumulative prednisolone dose. Thus, the patients who were given avacopan before day 30 from induction therapy were more likely to stop steroid administration at week 26 than those given it on day 30 or later (percentage of discontinuation of steroids; 52% vs. 33%) [1, 11]. In our case, avacopan was administered on hospital day 8 and prednisolone was successfully discontinued at week 22 while maintaining remission at week 52; hence, among the patients treated with ECMO and avacopan, our case is the first patient that achieved successful withdrawal of prednisolone within 6 months [2]. Furthermore, GTI-CWS and GTI-AIS observed at 26 weeks were comparable to those reported for Japanese patients in the ADVOCATE trial (44.9 ± 12.5 and 7.3 ± 8.3, respectively) [12]. These observations suggest that avacopan contributes to the steroid-sparing and minimizing its adverse effects.

The kidney is a major target for AAV, which causes pauci-immune crescentic glomerulonephritis. In our case, serum creatinine was slightly elevated on admission and the renal histology showed a feature of AAV (Fig. 3). Of note, a mild to moderate degree of proteinuria was observed and was reduced to a level less than 0.3 g/gCr on day 57 and thereafter (Fig. 2). The early reduction in proteinuria might be attributed to a relative mild renal injury. Alternatively, early initiation of avacopan treatment might help improve the disease activity [1, 2, 11, 13]; the ADVOCATE trial demonstrated that the early initiation of avacopan administration was associated with a shorter period to achieve nadir proteinuria (84 vs. 173 days, for avacopan initiation of < 30 days and ≥ 30 days from induction therapy, respectively) [1, 2].

To further explore the impact of early avacopan initiation, we compared our case with previously reported cases with DAH. While there is variability in age, ANCA subtype, and organ involvement, a common feature among these cases appears to be the early initiation of avacopan relative to disease onset (Table 2). Of note, a recent real-world study reported a median avacopan initiation time of 3.6 weeks (IQR: 2.1–7.7 weeks) [14]. Although direct comparison is limited due to differences in patient populations and disease severity, favorable clinical outcomes have been observed even in critically ill patients with DAH, including our case. These findings suggest that early initiation of avacopan may be beneficial in severe cases and warrant further investigation in larger cohorts.

**Table 2:** Comparison of disease characteristics and therapeutics

	Our case	Chalkia et al^2^ (n = 8)	Falde et al^3^ (n = 15)
Age (y/o)	31	64 [IQR 17–80]	66 [IQR 52–72]
ANCA subtype	MPO-ANCA	MPO-ANCA: n = 5 PR3-ANCA: n = 3	MPO-ANCA: n = 7 PR3-ANCA: n = 8
BVAS at diagnosis	18	Not reported	8 [IQR 5–10]
Organ Involvement	Lung, Kidney, ENT	Lung (n = 8), Kidney (n = 7), ENT (n = 5), NS (n = 2)	Lung (n = 15), Kidney (n = 9), ENT (n = 7), NS (n = 1)
Diffuse alveolar hemorrhage	(+)	(+): n = 7	(+): n = 15
ECMO implementation	(+)	(+): n = 1/(−): n = 7	(+): n = 0/(−): n = 15
Immunosuppressive treatment	Steroid pulse, Rituximab, Avacopan	Steroid pulse (n = 8), Rituximab (n = 7), Cyclophosphamide (n = 5), Avacopan (n = 8)	Steroid pulse (n = 12), Rituximab (n = 14), Cyclophosphamide (n = 3), Avacopan (n = 15)
Plasma exchange Number of sessions	3	7 [IQR 6.5–9.5] (n = 4)	4 [IQR 4–4] (n = 5)
Timing of avacopan initiation (hospital day)	Day 8	Day 6 [IQR 4–22.5]	Day 18 [IQR 2–24]
Steroid discontinuation	Day 156	Day 35 [IQR 32–37] (n = 5)^1^	Day 52 [IQR 26–114] (n = 11)^2^

*ANCA* antineutrophil cytoplasmic antibody, *MPO* myeloperoxidase, *PR3* proteinase 3, *BVAS* Birmingham Vasculitis Activity Score, *ENT* ear, nose, throat, *NS* nervous system, *ECMO* extracorporeal oxygenation therapy, *IQR* interquartile range

^1^Three patients continued at 1 year. Among them, one patient with ECMO continued steroid (prednisolone 25 mg) at 1 year

^2^Four patients continued during follow-up period

Finally, caveat is in order since our case received multiple medications and therapeutic modalities, which might overestimate the efficacy of avacopan in our case report. Thus, further in-depth studies are required to clarify the specific contribution of avacopan to clinical benefit.

In summary, we report a case of AAV presenting with severe respiratory failure requiring mechanical ventilation and ECMO support in which immunosuppressive drugs and avacopan as induction treatment offered successful remission without steroid maintenance therapy. Early use of avacopan, along with other immunosuppressives such as rituximab, may facilitate recovery with no relapse of alveolar hemorrhage and allow steroid withdrawal, even in the case of life-threatening respiratory failure.

## Data Availability

The datasets generated and/or analyzed during the current study are not publicly available due to limitations of ethical approval and anonymity constraints involving the patient data, but are available from corresponding author on reasonable request.
